# Integrating Xpert MTB/RIF for TB diagnosis in the private sector: evidence from large-scale pilots in Patna and Mumbai, India

**DOI:** 10.1186/s12879-021-05817-1

**Published:** 2021-01-28

**Authors:** Sarang Deo, Pankaj Jindal, Sirisha Papineni

**Affiliations:** 1 Indian School of Business, AC 3, L1, #3113, ISB Campus, Gachibowli, Hyderabad, 500032 India; 2 UCLA Anderson School of Management, Los Angeles, United States; 3 Harvard Medical School, Boston, United States; 4 World Health Partners, New Delhi, India

**Keywords:** Xpert, Tuberculosis, Diagnosis, Private sector

## Abstract

**Background:**

Xpert MTB/RIF (Xpert) has been recommended by WHO as the initial diagnostic test for TB and rifampicin-resistance detection. Existing evidence regarding its uptake is limited to public health systems and corresponding resource and infrastructure challenges. It cannot be readily extended to private providers, who treat more than half of India’s TB cases and demonstrate complex diagnostic behavior.

**Methods:**

We used routine program data collected from November 2014 to April 2017 from large-scale private sector engagement pilots in Mumbai and Patna. It included diagnostic vouchers issued to approximately 150,000 patients by about 1400 providers, aggregated to 18,890 provider-month observations. We constructed three metrics to capture provider behavior with regards to adoption of Xpert and studied their longitudinal variation: (i) Uptake (ordering of test), (ii) Utilization for TB diagnosis, and (iii) Non-adherence to negative results. We estimated multivariate linear regression models to assess heterogeneity in provider behavior based on providers’ prior experience and Xpert testing volumes.

**Results:**

Uptake of Xpert increased considerably in both Mumbai (from 36 to 60.4%) and Patna (from 12.2 to 45.1%). However, utilization of Xpert for TB diagnosis and non-adherence to negative Xpert results did not show systematic trends over time. In regression models, cumulative number of Xpert tests ordered was significantly associated with Xpert uptake in Patna and utilization for diagnosis in Mumbai (*p*-value< 0.01). Uptake of Xpert and its utilization for diagnosis was predicted to be higher in high-volume providers compared to low-volume providers and this gap was predicted to widen over time.

**Conclusions:**

Private sector engagement led to substantial increase in uptake of Xpert, especially among high-volume providers, but did not show strong evidence of Xpert results being integrated with TB diagnosis. Increasing availability and affordability of a technically superior diagnostic tool may not be sufficient to fundamentally change diagnosis and treatment of TB in the private sector. Behavioral interventions, specifically aimed at, integrating Xpert results into clinical decision making of private providers may be required to impact patient-level outcomes.

**Supplementary Information:**

The online version contains supplementary material available at 10.1186/s12879-021-05817-1.

## Background

Tuberculosis (TB) continues to be a major global health challenge resulting in more than 10 million new cases and 1.2 million deaths in 2018 [1]. Early and accurate diagnosis followed by successful treatment completion are essential pillars of the End TB strategy of the World Health Organization (WHO) [2]. The global fight against TB received a significant boost with the development of a new molecular diagnostic test, Xpert MTB/RIF (hereafter Xpert) [3]. It is significantly more accurate than smear microscopy, substantially faster than microbiological culture and can simultaneously detect rifampicin resistance [4, 5]. Models calibrated using data from early feasibility studies of Xpert showed that its large-scale adoption would significantly reduce disease burden and remain cost-effective [6–8]. Xpert was approved by WHO be used as the initial diagnostic test for the detection of TB and rifampicin resistance in adults and children. Following this, by 2016, more than 2000 Xpert instruments and more than 16 million cartridges had been procured in more than 130 countries [9, 10].

Recent evidence, however, suggests that the actual impact of Xpert may have fallen short of its potential due to a few key reasons. First, concessional pricing negotiated with the manufacturer was restricted to the public sector in high burden countries. As a result, private providers, who play a dominant role in the diagnosis and treatment of TB in many of these countries [11, 12], continued to maintain a high price thereby restricting patient access [10, 13]. Second, even with large-scale rollout of Xpert in some national TB programs (e.g., South Africa, Brazil), evidence regarding its impact on patient relevant outcomes is mixed [14–16]. It was found that Xpert, compared to smear microscopy, increased the number and proportion of microbiologically confirmed TB case notifications and reduced the delay in treatment initiation. However, it did not significantly increase overall case notifications and did not have a significant impact on patient-relevant health outcomes such as mortality, tuberculosis-related morbidity, and successful treatment completion. These mixed findings are believed to be driven by high levels of empirical treatment, which may be partially replaced by Xpert [17–21], and by poor adherence to diagnostic algorithms involving Xpert compared to those involving smear microscopy [22].

These insights from the public sector cannot be easily extrapolated to estimate the impact of Xpert among private providers in high burden countries. They often do not use smear microscopy and, instead, rely on clinical diagnosis using chest X-ray and even serological tests, despite lack of evidence regarding their accuracy [23]. Their adoption of new technologies and adherence to protocols, guidelines and algorithms, is also influenced by explicit or implicit economic incentives [24, 25].

In India, which has a fourth of the global TB cases, private providers are estimated to treat more than half of them [26]. Mounting evidence suggests that they follow suboptimal diagnostic practices, which deviate significantly from the national standards of TB care [27–31]. Revised National TB Control Program (RNTCP)‘s strategic plan for TB elimination recommends using specialized professional agencies for large-scale engagement of private providers [32]. This engagement model consists of mapping and networking private sector providers who interact with TB patients (treating clinicians, diagnostic facilities, and pharmacists); and providing a package of services to this network aimed to improve quality of care for patients. Services include continuous clinical and technical training of treating providers to improve their diagnostic and treatment practices as well as free access to high quality drugs and diagnostic tests such as Xpert for their patients [33]. Early evidence from pilots in two cities (Mumbai and Patna) suggests that this model can successfully engage a large number of private providers and substantially increase overall case notifications [34–36]. However, its impact on the uptake of high quality test such as Xpert and integration of its results with clinical decision making by private providers is not known.

In this study, we analyzed detailed programmatic data from these large-scale pilots to quantify changes in provider behavior over time with respect to their uptake of Xpert and utilization of its results for TB diagnosis. We also explored any heterogeneity across providers on these measures and operational factors associated with the heterogeneity.

## Methods

### Study setting and intervention

Our study was conducted in the cities of Patna (Bihar) and Mumbai (Maharashtra), where a large-scale private provider engagement program was launched in May 2014. The program was implemented by two separate non-governmental organizations (NGOs) that acted as a private provider interface agency (PPIA). In Patna, the program covered the entire urban population of 6.2 million with an annualized TB case notification rate of 350 per 100,000 whereas, in Mumbai, it covered a population of 12.8 million in 13 high burden wards with an annualized TB case notification rate of 340 per 100,000.

The program engaged a network of formal providers (medical practitioners qualified in allopathic medicine), informal providers (all other providers including those trained in indigenous medicine and those without any formal medical qualification), laboratories, and pharmacies within the private sector. The objective of this PPIA network was to improve the diagnosis, notification, and successful treatment completion of TB cases in the private sector through the provision of subsidized drugs and diagnostic tests. In Mumbai, both formal and informal providers could order a free chest X-ray (CXR), and formal providers could order a free Xpert at networked laboratories. In Patna, in addition to the above provisions, both formal and informal providers could also order free smear microscopy tests. Patients diagnosed with TB, either microbiologically or clinically, were initiated on treatment by formal providers and could access free anti-TB drugs at a network pharmacy. Diagnostic and treatment services were reimbursed by the PPIA program. PPIA program staff conducted Continuing Medical Education (CME) seminars as well as routine visits to train and sensitize network providers on Standards of TB care in India with particular emphasis on the utilization of Xpert for microbiological confirmation of TB.

In addition to diagnosis, the program facilitated collection and transportation of sputum samples from providers’ clinic to laboratories for Xpert testing thereby removing time and cost constraints for patients. Finally, the program staff also supported the providers to meet their legal obligation of notifying TB cases in the national database, Nikshay, and provided treatment adherence support to patients using a call center and field staff for household visits.

### Data

We obtained program data on diagnostic and treatment vouchers issued by providers over a period of 30 months from May 2014 to April 2017. Program data was collected from the PPIA programs and Nikshay database, which consisted of presumptive TB cases (defined as patients experiencing TB symptoms and require further investigation) and notified TB cases (defined as either microbiologically confirmed or clinically diagnosed TB cases that were reported into Nikshay with a treatment initiation date). For each diagnostic voucher, the dataset contained the name of the test ordered, the date of voucher’s issuance by a network provider, the date of its validation by a network laboratory, the date of availability of the test result along with unique identifiers for the provider and the patient. Test results were categorized as “Acid-fast Bacillus (AFB) seen/AFB not seen” for smear microscopy, “shadow/no-shadow” for CXR, and “*Mycobacterium tuberculosis* (MTB) detected/not detected” as well as “Rifampicin (RIF) resistance detected/not detected” for Xpert. Finally, for patients diagnosed with TB and initiated on treatment, the dataset contained the site. i.e., pulmonary/extrapulmonary as well as the date of treatment initiation. We excluded observations corresponding to informal providers because they were not allowed to issue vouchers for Xpert, which was the focus of our study. We also excluded observations during the first 6 months of the program, i.e., May 2014 to October 2014 to allow for stabilization of program activities and data collection processes.

### Analysis

We conducted our analysis in two stages. First, we aggregated voucher data at the level of unique patients and used it to calculate three measures to capture the adoption of Xpert by private providers and incorporation of its results into their clinical decision making. We defined “*Uptake”* as the proportion of all patients-- both presumptive and notified patients--for whom Xpert was ordered. “Uptake” did not distinguish the reason for Xpert use and was inclusive of all Xpert testing conducted, whether it was for upfront TB diagnosis or subsequent testing for rifampicin resistance during the treatment duration period. “Uptake” therefore signaled the providers’ willingness to include an Xpert test within his or her clinical algorithm.

In order to assess the interpretation and integration of Xpert results to arrive at a TB diagnosis specifically, we constructed two additional metrics. We defined *“Utilization for TB Diagnosis”* as the proportion of notified pulmonary TB cases who received a Xpert test for initial TB diagnosis. We classified an Xpert to be for *diagnostic* purpose if the date of issuing the test voucher was either before or up to 15 days after the date of treatment initiation of a TB patient. This timeframe corresponded to a diagnostic phase whereby a provider may decide to initiate or cease TB treatment based on Xpert results. Finally, we defined “*Non-adherence to negative results”* as the proportion of patients with a negative diagnostic Xpert result (MTB not detected), who were still subsequently initiated on TB treatment. This metric reflected the providers’ confidence in overriding a Xpert result based on clinical judgement and/or reliance on other diagnostics.

We calculated monthly values of these measures, separately for each program, and plotted them against time to capture temporal trends in aggregate provider behavior. Second, to understand the changes in behavior with respect to Xpert at the level of individual provider, we aggregated data at provider-month level. This yielded a panel dataset comprising 8154 observations for Patna and 10,726 observations for Mumbai. We formulated three multivariate linear panel regression models, one for each of the above three measures and estimated them separately for Mumbai and Patna programs. The outcome variables in these models were numerators of the three measures described earlier, aggregated at the provider-month level: (i) number of Xpert tests ordered, (ii) number of notified pulmonary TB cases with treatment initiation date after the date of availability of Xpert result, and (iii) number of notified TB cases with a negative Xpert result. The main predictor variables of interest in these models were the cumulative number of patients seen and the cumulative number of Xpert orders until the previous month, which capture the learning-by-doing effect, i.e., providers becoming more familiar and comfortable with the test because of their prior experience of using it. In each of the three models, we controlled for the denominators of the corresponding measure aggregated at the provider-month level: (i) number of patients, (ii) number of notified pulmonary TB cases who was ordered a diagnostic Xpert, and (iii) number of patients that received a negative Xpert (MTB not detected) result. All models included fixed effects for providers and month to account for provider- and time-invariant factors, respectively. We used two-way clustering of standard errors to account for possible correlation in the errors between different providers within the same month and between different months within the same provider. To check the robustness of our results, we considered alternate model specifications that used a continuous variable for providers’ duration of engagement with the program instead of a fixed effect for month.

To understand the impact of patient volume and time on provider behavior, we used estimates from these models (Supplementary Text 1) to predict the four outcome variables for a representative high and low volume provider as follows. First, we categorized providers as high and low volume based on whether the total number of patients seen by them belonged to the top quartile (75th to 100th percentile) or the second quartile (50th to 75th percentile), separately for Mumbai and Patna. For each category, we considered a hypothetical representative provider for whom the value of predictor variables was equal to the monthly averages within each category and used the coefficient estimates of the four models to predict the four outcome variables for these representative high and low volume providers. We plotted these predicted values against time spent in the program, along with 95% prediction intervals, separately for Patna and Mumbai.

We performed all the statistical analyses using R programming language in integrated development environment of RStudio with model estimation done using the package *lfe* and organization of results done using the package *stargazer*.

## Results

Table 1 displays the summary statistics for the study period from November 2014 to April 2017. A total of 67,308 patients (both presumptive and notified) were registered by 501 formal providers in Patna and 77,174 patients were registered by 1428 formal providers in Mumbai. Of these patients, 35,262 and 36,405 were notified TB cases in Patna and Mumbai respectively. A total of 21,233 Xpert tests were ordered in Patna, and 40,093 Xpert tests were ordered in Mumbai over the study period. Total number of rifampicin resistant TB cases detected was 889 in Patna and 3482 in Mumbai. Of the formal providers registering patients, 429 providers in Patna and 1078 providers in Mumbai ordered at least one Xpert test during the study period.

**Table 1:** Description of the dataset (November 2014 to April 2017)

	Patna	Mumbai
Number of patients registered in PPIA by formal provider	67,308	77,174
Number of Xpert tests ordered	21,233	40,093
Number of notified TB cases	35,262	36,405
Number (%) microbiologically confirmed cases	5310 (15%)	12,960 (36%)
Number of pulmonary notified TB cases	23,625	31,529
Number of rifampicin resistant cases detected with Xpert	889	3482
Number of formal providers that registered patients in PPIA	501	1428
Number (%) of formal providers that ordered at least one Xpert	429 (86%)	1078 (75%)

**Figure 1:**
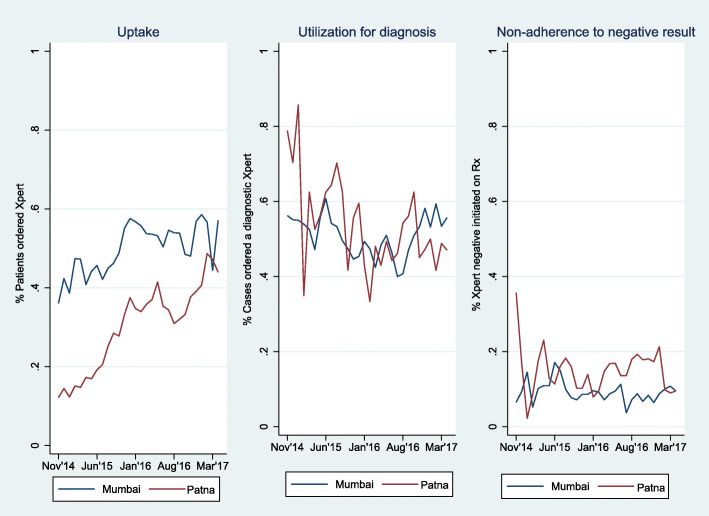
**Fig. 1** Adoption of Xpert and integration of its results in dignosis by providers over time

Figure 1 shows uptake, utilization for diagnosis, and non-adherence to negative results over the study period. Xpert uptake from the first six-month reporting period to the last six-month reporting period increased from 14.7 to 44.4% in Patna and 42.9 to 54.7% in Mumbai. Utilization of Xpert as diagnostic was variable without a clear programmatic trend, ranging from 57.6 to 46.8% in Patna and from 52.7 to 56.1% in Mumbai. Non-adherence to negative results remained relatively consistent over time with an overall 13.9% of Xpert MTB/RIF negative patients initiated on treatment in Patna and 8.8% of Xpert negative patients initiated on treatment in Mumbai.

Table 2 contains the estimates of the four regression models that capture the impact of prior experience and learning-by-doing on adoption of Xpert and its results for clinical decision making by providers. We found that number of cumulative patients seen until the previous month was positively associated with Uptake in Mumbai (0.018; *p*-value< 0.001). It was negatively associated with Utilization for diagnosis in Mumbai (− 0.002; *p*-value< 0.1) and positively associated with non-adherence to negative results in Patna (0.001; *p*-value< 0.001). Similarly, number of cumulative Xpert tests ordered until the previous month was positively associated with Uptake in Patna (0.033; *p*-value< 0.001) and with Utilization for diagnosis in Mumbai (0.006; *p*-value< 0.01). In terms of magnitude, associations were strongest for Uptake. Additional 100 cumulative patients seen until the previous month resulted in 1.8 additional Xpert tests ordered in the current month in Mumbai and additional 100 cumulative Xpert tests ordered until the previous month resulted in 3.3 additional Xpert tests in the current month in Patna. Most associations for Non-adherence to negative results were weak in magnitude, in a direction opposite to expectation, or not statistically significant.

**Table 2:** Estimates of the impact of provider specific factors on Xpert adoption

	Uptake		Utilization for diagnosis		Non-adherence to negative results	
	Patna	Mumbai	Patna	Mumbai	Patna	Mumbai
Cumulative patients seen till previous month (SE)	− 0.002 (0.004)	0.018^**^ (0.008)	0.001 (0.0004)	−0.002^*^ (0.001)	0.001^***^ (0.0003)	0.0004 (0.001)
Cumulative number of Xpert ordered till previous month (SE)	0.033^***^ (0.013)	−0.020 (0.014)	−0.001 (0.001)	0.006^**^ (0.002)	−0.0004 (0.001)	0.0003 (0.001)
Number of patients seen this month (SE)	0.306^***^ (0.074)	0.494^***^ (0.031)				
Number of notified cases with a diagnostic Xpert (SE)			0.551^***^ (0.045)	0.508^***^ (0.036)		
Number of pulmonary notified TB cases						
Number of patients with negative Xpert result (SE)					0.015 (0.010)	0.077^***^ (0.008)
Observations	8154	10,726	8154	10,726	8154	10,726
Adjusted R^2^	0.711	0.898	0.821	0.836	0.436	0.488

Note: All models include provider and month fixed effects. *p < 0.1; **p < 0.05; ***p < 0.01

**Figure 2:**
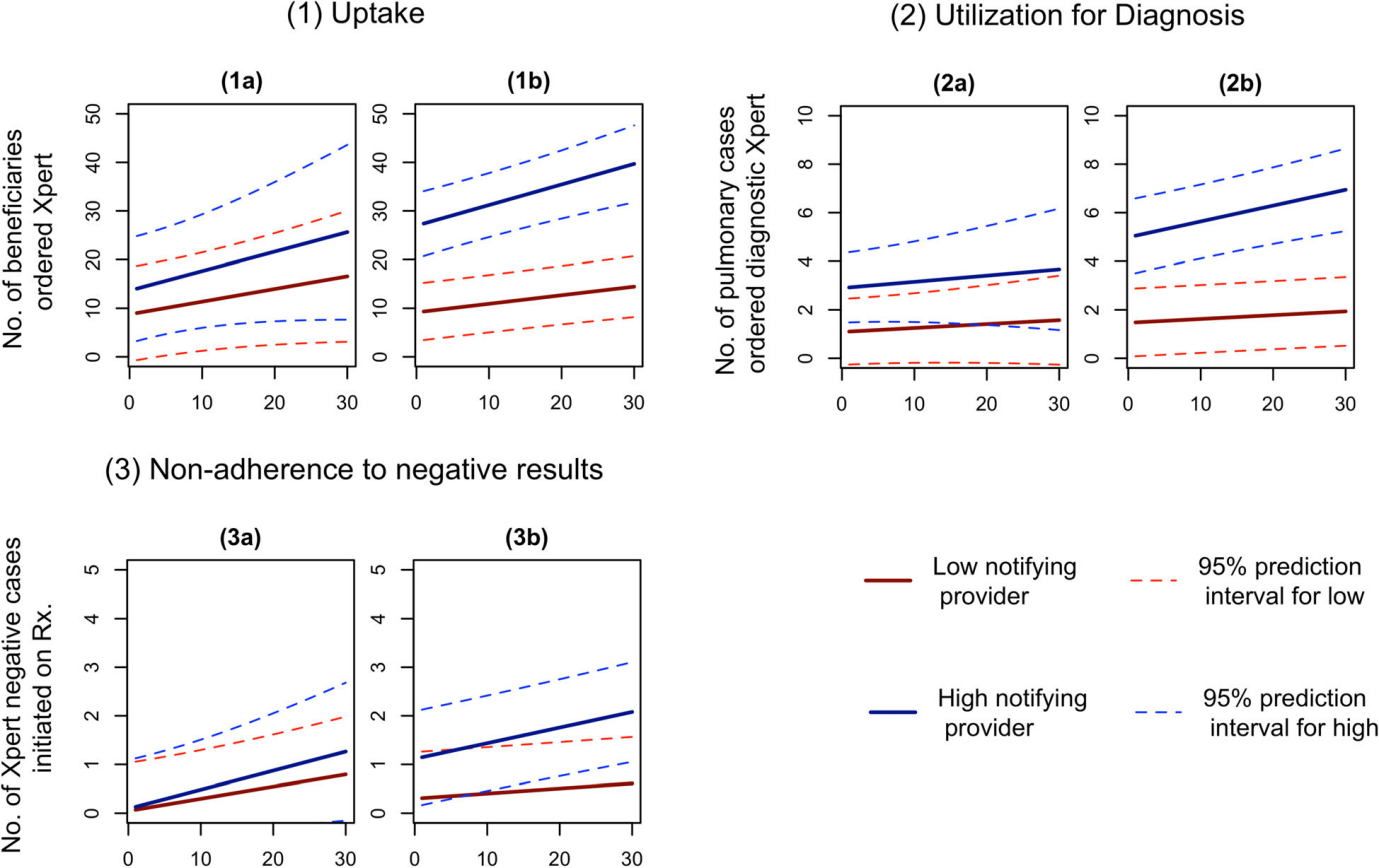
**Fig. 2** Predicted adoption of Xpert and integration of results in diagnosis by low and high volume providers

Figure 2 displays the change in predicted outcomes for a representative high and low volume provider with increasing duration of their engagement with the program (measured in months). In Patna, Xpert uptake was predicted to have increased by approximately 85% both for high volume provider (from 14.04 to 25.62 per month) and low volume provider (from 9.01 to 16.57 per month), but 95% prediction intervals were overlapping for the two categories. In Mumbai, the difference between the two categories was more prominent; uptake was predicted to increase by 45% for high volume providers (27.38 to 39.68 per month) and 55% for low volume providers (9.34 to 14.44 per month). Yet, the gap between the two categories was predicted to have widened over the course of the program engagement. Similar patterns were observed for utilization for diagnosis, where the gaps between high volume and low volume providers were predicted to have widened in both cities with a larger effect in Mumbai compared to Patna. Utilization for diagnosis increased by 25% (2.93 to 3.67 per month) and 43% (1.10 to 1.57 per month) for low- and high-volume providers, respectively. In Mumbai, the increase was 38% (5.05 to 6.94 per month) and 31% (1.48 to 1.94 per month), respectively. Non-adherence to negative results was predicted to decrease, i.e., fewer number of patients with negative Xpert results initiated on treatment for both cities but the difference across low and high-volume providers was not significant and the absolute numbers were small.

## Discussion

Xpert has been widely regarded as a game changer in the global fight against TB, but its impact on case finding and management in high burden countries is likely to be constrained by low adoption by private providers. Although broader uptake of Xpert can have implications on detection of rifampicin resistance, utilization of the tool as an initial diagnostic poses challenges in the private sector. Existing evidence regarding its impact is primarily from the public sector, and studies examining the integration of Xpert into clinical decision making are limited. Dialogue on scaling Xpert is largely focused on resource and financial constraints without sufficient regard to provider behavior change even if feasibility conditions are met. In this study, we used detailed programmatic data at the level of individual private providers to analyze changes in their behavior with respect to adoption of Xpert. We found that the uptake of Xpert increased substantially over time. The exclusion of first 6 months of program data had little effect on overall Xpert uptake; a cumulative 249 Xpert tests were ordered during this period, comprising of less than 1 % of the total Xpert volume. Although the uptake of Xpert increased, its upfront utilization for TB diagnosis and the initiation of TB treatment after a Xpert negative result did not exhibit favorable programmatic changes over time. Xpert was more likely to be used as either an “add-on” tool to validate the diagnosis already made by the treating provider or selectively used for drug susceptible testing. We found significant heterogeneity in behavior across providers within each city; high volume providers exhibited greater change in their behavior compared to low volume ones. Finally, we also found substantial difference in provider behavior across the two cities, likely reflecting the difference in the composition of provider base in these cities, their baseline awareness and practices, and local TB epidemiology.

In Patna, where Xpert was relatively unknown among private providers before the PPIA program, its uptake increased more than threefold over the course of study period. In Mumbai, Xpert uptake increased almost 1.2 times, likely due to a higher baseline owing to higher awareness of Xpert among private providers. Previous studies on Xpert adoption in India have focused on public sector. A demonstration study in 18 sub-district level units in the national program found that implementation of Xpert was associated with increases in both notification rates and proportion of microbiologically confirmed pulmonary TB cases, but the study did not involve a control group so establishing causality of Xpert implementation was not possible [37]. Similar increases in case notification have been observed from implementation of Xpert among pediatric and HIV patients in the public sector [38, 39]. In the private sector, creating a consortium of private laboratories was found to increase Xpert testing volumes of private providers during the same time as our study period [40]. However, this approach differs from the program studied here in two important aspects. First, it focused only on improving Xpert testing and was not embedded in a larger TB treatment and management program. Second, it provided Xpert testing at a subsidized price whereas it was completely free for patients in the PPIA program studied here. These differences are likely to translate into substantial differences in the magnitude of increase in uptake of Xpert. Another program, which engaged more than 400 private and public sector providers across four cities reported an increase in the uptake of Xpert for diagnosing pediatric TB when provided free of cost to patients [41]. However, results from that study are not directly comparable to ours due to its focus on pediatric patients. Further, none of these studies conducted a detailed provider-level analysis and analyzed the heterogeneity across providers. Similarly, they did not analyze how the results from Xpert were utilized by providers in clinical decision making.

In both cities, increasing Xpert utilization for TB diagnosis was a bigger challenge (compared to uptake alone) with no clear programmatic trend. Moreover, the fraction of notified cases with a negative Xpert result also remained reasonably unchanged over the course of the program. Combined with the increased uptake of Xpert, these findings indicate that a providers’ test ordering behavior does not reflect the actual incorporation of test results in their clinical decision making. Private providers in India are known to rely on clinical diagnosis and empiric treatment initiation [42]. Thus, providers may have used Xpert results when they confirmed their clinical judgment but not when the results conflicted with their judgment. This is in line with previous studies that have found that physicians are prone to confirmation bias, i.e., they selectively choose evidence that aligns with their prior beliefs and ignore that which is counter [43, 44]. We calculated positivity rate of Xpert (defined as the proportion of diagnostic Xpert results that were positive) to check the possibility of this behavior. In Mumbai, positivity rate was 46.7% at the beginning of the study period and declined to 32.6% in the last reporting period. In Patna, it remained relatively stable around 30–35% throughout the study period, which was significantly greater than the 5–10% positivity rate for smear microscopy (Supplementary Figure 1). The decrease in positivity observed in Mumbai is likely due to a shift from using Xpert primarily for the detection of rifampicin resistance (given the higher baseline knowledge of Xpert as a test for drug resistance), and subsequent expansion of Xpert use for initial TB diagnosis. The PPIA program staff in Mumbai also sensitized and monitored providers on the specific use of Xpert as an initial TB diagnostic whereas the Patna PPIA program sensitized and monitored providers on the broader uptake of Xpert without dictating its utilization. The messaging and frequency of interactions between provider and field staff have a potential to influence provider diagnostic behavior. Optimized strategies on such behavior change interventions over sufficient time can shift Xpert utilization.

Prior studies from other parts of the world have found that scale-up of Xpert in public programs increased the proportion of microbiologically confirmed cases but did not consistently increase the total number of notified cases. In fact, rollout of Xpert in South Africa’s national program actually decreased case notifications by 12–19% and rate of empirical treatment decreased by about 50% and took about 3 years after the launch of roll-out to stabilize [21]. Similarly, rollout of Xpert in Nepal reduced the annual case notification rate for pulmonary TB by 8.5% even though microbiologically confirmed TB notifications increased by 15.2% [45]. These findings suggest that Xpert may have helped to reduce overdiagnosis due to clinical diagnosis. In contrast, rollout of Xpert in Guatemala found more than 40% increase in TB case notification [46]. The difference in these findings may be attributable to the difference in prevalence of empirical treatment and quality of clinical diagnosis across these contexts. Overall, the implications of our findings are quite similar. They challenge the simplistic view that improved availability of a technically superior diagnostic tool such as Xpert will fundamentally change how TB is being diagnosed and treated. As a result, it is possible that the scale-up of this intervention, as-is, may not positively impact patient-relevant outcomes such as TB-related morbidity and mortality.

However, despite the similar conclusion, it is important to note key differences in the underlying factors that led to this conclusion in our setting compared to prior studies. First, usage of smear microscopy among private providers in our context was substantially lower compared to public programs in those studies. Second, providers in our data were not required to follow fixed diagnostic algorithms, given that there is no uniform diagnostic algorithm that is practiced in the private sector. Prior research reflects a diverse use of diagnostic algorithms including clinical diagnosis without TB diagnostic tests or clinical diagnosis with a CXR suggestive of TB, with low utilization of microbiological tests and high rates of empirical treatment across providers [27, 47]. In fact, providers ordered various combination of tests and investigations that were subsidized by the program, i.e., CXR and Xpert in Mumbai and CXR, Xpert and smear microscopy in Patna. Descriptive analysis of these combinations suggested that providers may have used Xpert as an “add-on” test rather than as “replacement” (Supplementary Figure 2). Third, provider behavior was influenced by continuous provider follow-ups and sensitizations by program staff on the use of Xpert with the purpose of shifting provider behavior.

Prior experience of providers (measured either in terms of cumulative volume of patients or cumulative volume of Xpert tests conducted) had varying degrees of association with outcome variables of interest with the strongest association in Xpert uptake across both cities. Predictions based on provider-level model estimates showed that the gap in the uptake of Xpert and its utilization for diagnosis between low volume and high-volume providers would have widened over the course of the program, especially in Mumbai. In other words, engaging with high volume providers may provide increasing returns on investment over time. Prior studies have found that provider and hospital volumes are associated with better patient outcomes [48, 49]. However, the mechanisms proposed to explain these associations are (i) learning curves or the notion that practice makes perfect, which has been observed in other industries such as aircraft and chemical manufacturing [50], and (ii) selective referrals. The relative magnitude of these effects has been shown to vary across multiple surgical procedures and specialties, perhaps in line with intuition rooted in medical knowledge [51]. In contrast, our field experience suggests that our findings may be driven by high volume providers finding it easier and faster to form a new prescription habit, which is known to be a major component in physician decision process [52]. In addition, higher volume also implies that the time lag between obtaining Xpert results for one patient and ordering it for the next patient is shorter thereby enabling more effective learning from successes and failures [53].

Results from this study have important implications for private sector engagement for TB control in India. The Indian National Strategic Plan (NSP) of 2017–2025 has ambitious targets to expand private sector engagement to improve quality of care in the private sector, including the procurement of Xpert testing for diagnostic capacity at every district in the country; revision of national guidelines to include Xpert MTB/RIF in diagnostic algorithms; and strengthen implementation research to demonstrate resource optimization for revised diagnostic algorithms [32]. The PPIA model is being scaled up to over 50 cities in India through the Joint Effort for Elimination of TB (JEET) supported by Global Fund as well as state contracting of private agencies (similar to PPIA) to achieve NSP objectives. Against this backdrop, our findings provide useful guidance for designing provider engagement strategies for implementing agencies. First, targeting high volume providers for engagement will not only lead to greater number of cases being notified but also result in greater impact in terms of diagnostic behavior change at the program level. Second, the availability and affordability of a superior diagnostic test does not guarantee the desired incorporation into diagnostic algorithms. Behavioral change interventions with optimized communication and messaging to providers, emphasizing integration of Xpert results beyond uptake only, are required. Provider-level follow-ups with more individualized messaging addressing the underlying reasons for poor Xpert utilization can drive behavioral change as compared to large CME seminars alone. Provider behavior metrics can be routinely monitored along with creative motivational nudges such as comparative score cards (i.e. scoring Xpert utilization across providers), which have shown to impact behavior of healthcare providers in other contexts [54, 55].

The main strength of our study is access to granular operational data over a long period of time. This allows us to observe within-provider behavior changes and thus avoid the need to control for confounders that can drive aggregate program-level outcomes. The other strength of our study is that the program involved free provision of Xpert thereby removing financial barriers to its adoption. Thus, our results are indicative of purely behavioral barriers in adoption of Xpert and its use in clinical decision making.

Our study also has several limitations due to the use of programmatic data. First, we did not have access to information on patients seen by the providers who were not issued any diagnostic voucher. As a result, our estimates of Xpert usage and penetration are likely to be biased upwards. However, in Patna, it is unlikely that providers and patients had access to Xpert outside of the PPIA program, given that it was a newly introduced test in the private sector. Even in Mumbai, where private labs did offer Xpert, the market price of the test was prohibitively high and penetration low [40]. Second, our study was not designed to establish causal link between the program and adoption of Xpert, and it did not involve any control set of providers for comparison. However, there were no other major changes in the TB diagnosis and treatment landscape in these cities that could have led to these changes in Xpert uptake and utilization for diagnosis. Third, we were unable to delineate exact mechanisms and components of the PPIA program that may have contributed to provider behavior change. Interactions with providers suggest that high-quality service delivery and routine provider surveillance were critical success factors. Fourth, our findings cannot be easily generalized to other cities in India. Mumbai has a large DR-TB epidemic and providers are more aware of new molecular tests compared to other cities. On the other hand, private providers in Patna are likely to be at the other end of the awareness spectrum. Fifth, we could not directly observe and measure the intent of the providers in ordering these tests and using their results in the diagnosis process along with the full range of diagnostic tests that are typically ordered (outside of the PPIA diagnostic services offered). Therefore, we were not able to fully analyze how Xpert was integrated into existing diagnostic decision making process of providers, which is known to be complex [42, 56]. Future qualitative and quantitative studies should further explore these nuanced effects of a new technology on providers’ decision making. Finally, the study could not comment on whether Xpert improved TB case notification to program, microbiological confirmation, treatment initiation, treatment completion, or TB mortality, as it was only designed to examine provider-level behavior. The study was only designed to study provider-level behavior with Xpert (uptake and utilization), which can subsequently explain patient-level outcome measures.

## Conclusions

In this study, we found that private sector engagement led to substantial increase in uptake of Xpert, especially among high-volume providers. However, we did not find strong evidence for integration of results of Xpert with the eventual TB diagnosis of the patient. These findings suggest that increasing availability and affordability of a technically superior diagnostic tool (e.g., Xpert), by themselves, may not be adequate to improve TB diagnosis and treatment in the private sector. Policy makers and program managers may need to supplement the availability of tools with behavioral interventions aimed at integrating Xpert results into clinical decision making of private providers to impact patient-level outcomes.

## Supplementary Information

**Additional file 1.**

**Additional file 2.**

**Additional file 3.**

## Data Availability

The datasets generated and/or analyzed during the current study are not publicly available due to their proprietary nature and sensitivity owing to commercial considerations but are available from the corresponding author on reasonable request.

## Abbreviations

TB: Tuberculosis
RNTCP: Revised National TB Control Program
PPIA: Private provider interface agency
CME: Continuing Medical Education
CXR: Chest X-ray
AFB: Acid-fast Bacillus
MTB: *Mycobacterium tuberculosis*
RIF: Rifampicin
