# Supplementary material for: Integrating Xpert MTB/RIF for TB diagnosis in the private sector: evidence from large-scale pilots in Patna and Mumbai, India
Source: BMC Infect Dis. 2021 Jan 28;21:123. doi: 10.1186/s12879-021-05817-1 (PMC7844908; doi:10.1186/s12879-021-05817-1)
Supplement: Supplementary file 1 — Additional file 1. [file 12879_2021_5817_MOESM1_ESM.pdf]

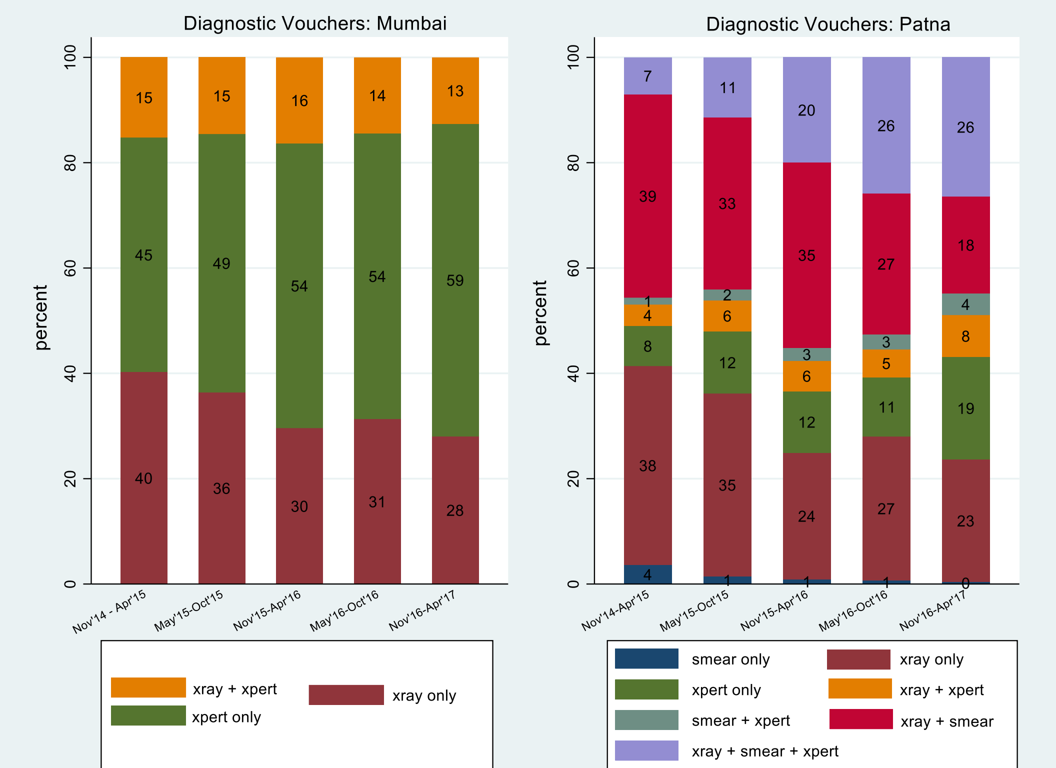
Diagnostic Vouchers: Mumbai
percent
0
20
40
60
80
100
40
45
15
36
49
15
30
54
16
31
54
14
28
59
13
Nov'14 - Apr'15
May'15-Oct'15
Nov'15-Apr'16
May'16-Oct'16
Nov'16-Apr'17
xray + xpert
xpert only
xray only
Diagnostic Vouchers: Patna
percent
0
20
40
60
80
100
4
38
8
4
1
39
7
1
35
12
6
2
33
11
1
24
12
6
3
35
20
1
27
11
5
3
27
26
0
23
19
8
4
18
26
Nov'14-Apr'15
May'15-Oct'15
Nov'15-Apr'16
May'16-Oct'16
Nov'16-Apr'17
smear only
xray only
xpert only
xray + xpert
smear + xpert
xray + smear
xray + smear + xpert
