# Supplementary figures and images for: Integrating Xpert MTB/RIF for TB diagnosis in the private sector: evidence from large-scale pilots in Patna and Mumbai, India
Source: BMC Infect Dis. 2021 Jan 28;21:123. doi: 10.1186/s12879-021-05817-1 (PMC7844908; doi:10.1186/s12879-021-05817-1)

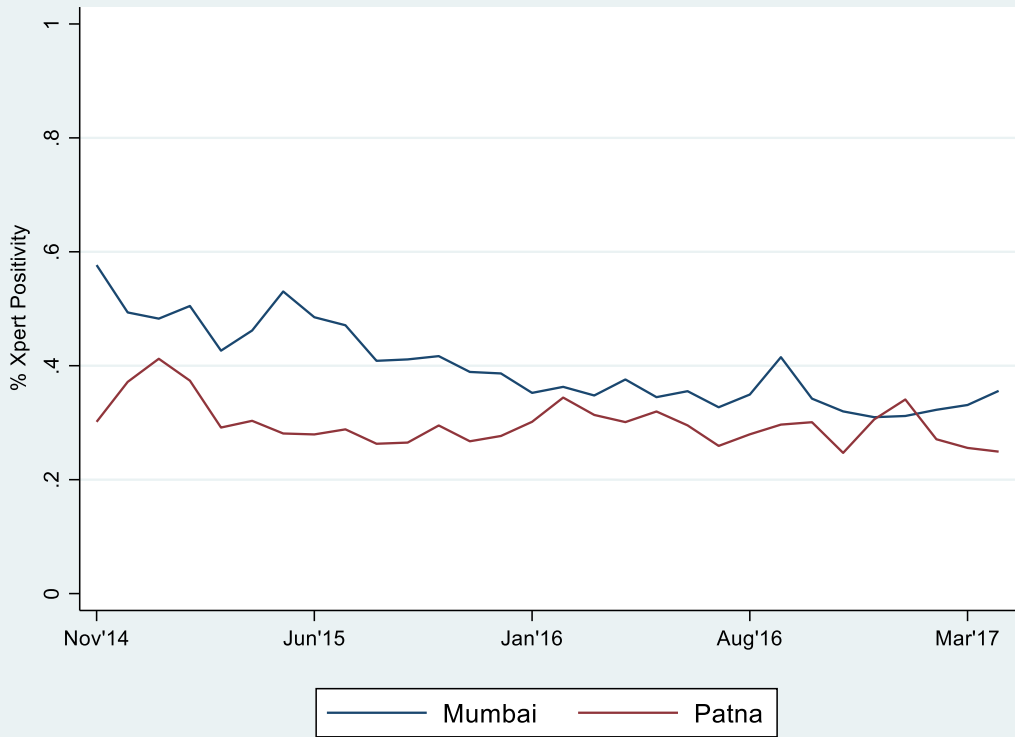

Supplement: Supplementary file 2 — Additional file 2. [file 12879_2021_5817_MOESM2_ESM.pdf]
