# Supplementary material for: Integrating Xpert MTB/RIF for TB diagnosis in the private sector: evidence from large-scale pilots in Patna and Mumbai, India
Source: BMC Infect Dis. 2021 Jan 28;21:123. doi: 10.1186/s12879-021-05817-1 (PMC7844908; doi:10.1186/s12879-021-05817-1)
Supplement: Supplementary file 3 — Additional file 3. [file 12879_2021_5817_MOESM3_ESM.docx]

# Model specifications

Following is a formal specification of the main models for the four models (i = index for provider, t = index for month, δ _i_ = provider fixed effects, and γ _t_ = time fixed effects):

**1. Xpert Uptake:**

(Number of Xperts ordered) _i,t_ = β _0_ + δ _i_ + γ _t_ + β _1_ (Number of beneficiaries seen) _i,t_ + β _2_ (Cumulative number of beneficiaries seen till t-1 month) _i,t_ + β _3_ (Cumulative number of Xperts ordered till t-1 month) _i,t_ + ε _i,t_

**2. Utilization of Xpert MTB/RIF for TB diagnosis:**

(Number of non-MDR beneficiaries with an Xpert result within 15 days before treatment initiation) _i,t_ = β _0_ + δ _i_ + γ _t_ + β _1_ (Number of non-MDR beneficiaries with an Xpert result within 15 days of treatment initiation) _i,t_ + β _2_ (Cumulative number of beneficiaries seen till t-1 month) _i,t_ + β _3_ (Cumulative number of Xperts ordered till t-1 month) _i,t_ + ε _i,t_

**3. Utilization of Xpert MTB/RIF for drug susceptibility testing (DST):**

(Number of all pulmonary notified cases started on treatment with an Xpert ordered) _i,t_ = β _0_ + δ _i_ + γ _t_ + β _1_ (Number of all pulmonary notified cases started on treatment) _i,t_ + β _2_ (Cumulative number of beneficiaries seen till t-1 month) _i,t_ + β _3_ (Cumulative number of Xperts ordered till t-1 month) _i,t_ + ε _i,t_

**4. Confidence in Xpert MTB/RIF:**

(Number of non-MDR beneficiaries started on treatment within 15 days after a negative Xpert result) _i,t_ = β _0_ + δ _i_ + γ _t_ + β _1_ (Number of non-MDR TB beneficiaries with a negative Xpert result) _i,t_ + β _2_ (Cumulative number of beneficiaries seen till t-1 month) _i,t_ + β _3_ (Cumulative number of Xperts ordered till t-1 month) _i,t_ + ε _i,t_
